# Poor Internal Jugular Venous Outflow Is Associated with Poor Cortical Venous Outflow and Outcomes after Successful Endovascular Reperfusion Therapy

**DOI:** 10.3390/brainsci13010032

**Published:** 2022-12-23

**Authors:** Wenjin Shang, Kaiyi Zhong, Liming Shu, Zhuhao Li, Hua Hong

**Affiliations:** 1 Department of Neurology, The First Affiliated Hospital, Sun Yat-sen University, Guangdong Provincial Key Laboratory of Diagnosis and Treatment of Major Neurological Diseases, National Key Clinical Department and Key Discipline of Neurology, Guangzhou 510080, China; 2 Department of Neurology, The Second Affiliated Hospital, Guangzhou Medical University, Guangzhou 510260, China; 3 Department of Radiology, The First Affiliated Hospital, Sun Yat-sen University, Guangzhou 510080, China; 4 Department of Geriatrics, The First Affiliated Hospital, Sun Yat-sen University, Guangzhou 510080, China; 5 Health Management Center, The First Affiliated Hospital, Sun Yat-sen University, Guangzhou 510080, China

**Keywords:** angiography, endovascular reperfusion therapy, prognosis, stroke, venous flow

## Abstract

Many patients show poor outcomes following endovascular reperfusion therapy (ERT), and poor cortical venous outflow is a risk factor for these poor outcomes. We investigated the association between the outflow of the internal jugular vein (IJV) and baseline cortical venous outflow and the outcomes after ERT. We retrospectively enrolled 78 patients diagnosed with an acute anterior circulation stroke and successful ERT. Poor IJV outflow on the affected side was defined as stenosis ≥50% or occlusion of ipsilateral IJV, and poor outflow of bilateral IJVs was defined as stenosis ≥50% or occlusion of both IJVs. Poor cortical venous outflow was defined as a cortical vein opacification score (COVES) of 0 on admission. Multivariate analysis showed that poor outflow of IJV on the affected side was an independent predictor for hemorrhagic transformation. The poor outflow of bilateral IJVs was an independent risk factor for poor clinical outcomes. These patients also had numerical trends of a higher incidence of symptomatic intracranial hemorrhage, midline shift >10 mm, and in-hospital mortality; however, statistical significance was not observed. Additionally, poor IJV outflow was an independent determinant of poor cortical venous outflow. For acute large vessel occlusion patients, poor IJV outflow is associated with poor baseline cortical venous outflow and outcomes after successful ERT.

## 1. Introduction

Endovascular reperfusion therapy (ERT) is superior to standard medical care for stroke patients with acute anterior circulation occlusion, as demonstrated by several large randomized controlled trials [1,2,3]. However, severe complications can still occur, such as hemorrhagic transformation (HT) and cerebral edema, increasing medical costs and delaying rehabilitation [4]. Furthermore, the functional dependence rate is 51–62%, and the mortality rate is 15.3–19% 90 days post-treatment [1,2,3].

In most clinical studies, arterial collaterals are considered a key factor in determining treatment strategy and prognosis [5,6]. However, in clinical practice, it is not uncommon for ERT patients with good arterial collaterals to have serious complications and poor outcomes. Increasingly, evidence shows that collateral circulation is an overall process from arteries to veins, and the increased arterial blood flow requires sufficient downstream venous collaterals [7,8,9,10]. Even a single cortical vein occlusion can cause a decrease in regional cerebral perfusion and brain edema [11]. Recently, a number of clinical studies have confirmed that favorable cortical venous outflow is associated with favorable tissue-level collaterals, a lower incidence of HT, and better functional outcomes in patients who underwent ERT [12,13,14,15].

However, the anatomy and drainage area of cortical veins varies considerably, which creates more stringent requirements for researchers or clinicians [8]. In addition to cortical veins, an abnormal outflow of the deep cerebral venous system also seems to be associated with poor functional outcomes after ERT [16,17]. Through sinus confluences and transverse-sigmoid sinuses, the superficial and deep cerebral venous systems drain into the internal jugular vein (IJV), which drains more than two-thirds of the cerebral blood flow [18]. Moreover, IJV is anatomically constant, easy to locate, and is the primary drainage pathway of the area supplied by the middle cerebral artery (MCA) [7,8,18].

Hypoplasia often occurs in the transverse sinus (TS), and IJVs can be compressed by muscle, bony structure, or the carotid sinus in many places approaching the superior vena cava [9,19,20,21]. IJV stenosis can cause a severe increase in venous pressure and intracranial pressure, which would decrease after the IJV stenosis is relieved [20,22,23]. The poor outflow of TS and IJV was found to be associated with midline shift and early fatal brain edema in stroke patients without ERT [19,24]. This association has also been reported in patients with meningiomas and intracerebral hemorrhages [23,25]. However, the relationship between the outflow profile of TS and IJV and ERT outcomes has not been investigated. In this retrospective cohort study of patients undergoing ERT for acute anterior circulation stroke, we hypothesized that poor outflow of TS and IJV is related to poor baseline cortical venous outflow and would correlate with poor imaging and function outcomes.

## 2. Materials and Methods

### 2.1. Patients

We retrospectively reviewed the data of consecutive patients diagnosed with an acute ischemic stroke treated with ERT in our institution from November 2017 to March 2022. The institutional ethics committee approved the study, and all clinical investigations were conducted according to the Declaration of Helsinki or comparable ethical standards.

Patients were included if they (1) had an acute or near occlusion of an internal carotid artery (ICA) or MCA; (2) underwent ERT within 24 h from symptom onset with successful recanalization defined as modified thrombolysis in cerebral ischemia ≥ 2b; (3) had a pre-stroke modified Rankin Scale (mRS) score of ≤2; and (4) had complete clinical and imaging data during hospitalization. Patients with a pre-stroke mRS score of ≥3 and incomplete clinical and imaging data were excluded.

### 2.2. Image Analysis

All patients underwent head and neck CT angiography (CTA) on admission and a follow-up non-contrast head CT (NCCT) within 24–48 h of ERT. The number of reexaminations was determined according to the patients’ clinical symptoms. A 320-detector row 640-slice cone-beam multidetector CT scanner (Aquilion One, Toshiba Medical Systems, Otawara, Tochigi, Japan) was used for imaging. A non-contrast scan of the cervical vessels covering the aortic arch to the middle cranial fossa was performed first, followed by a whole-brain NCCT in wide-volume mode (five rotations with a 4 cm width per detector). After NCCT, 40 mL of contrast agent (Ultravist 370; Bayer HealthCare, Berlin, Germany) was administrated (5 mL/s) chased by 40 mL of saline (acquisition parameters: 120 kV, 112 mAs, total collimation width: 16 cm); 19 pulse rotation scanning points were collected within 55 s. Next, scans of the carotid artery and delayed phases were acquired by administrating 40 mL of contrast agent (5 mL/s) followed by 40 mL of saline (acquisition parameters: 120 kV, 112 mAs, total collimation width: 16 cm, slice thickness = 0.5 mm). 

Two authors, both with over three years of experience in neuroimaging, assessed the imaging data independently, blinded to the clinical data. TS hypoplasia was defined as a degree of stenosis at least 50% greater than that on the contralateral side [26]. We evaluated the outflow of IJV (including the brachiocephalic vein) based on the standards of Zaharchuk et al. [27]: (1) favorable outflow = normal or mild flattening of IJV, stenosis < 50%; (2) poor outflow = moderate–severe flattening or no visualization of IJV, stenosis ≥ 50%, or occlusion. The outflow of bilateral IJVs was further classified into three grades: (1) favorable outflow = normal or stenosis < 50% of bilateral IJVs; (2) intermediate outflow: normal or stenosis < 50% of one IJV, and stenosis ≥ 50% or occlusion of the other IJV; (3) poor outflow = stenosis ≥ 50% or occlusion of bilateral IJVs. Cortical venous outflow was evaluated by cortical vein opacification score (COVES) on the original head CTA images on admission [13]. COVES assigned 0–2 points to each of the three cortical veins according to the degree of opacification of the superficial middle cerebral vein, sphenoparietal sinus, and vein of Labbe (0: invisible, 1: moderate, 2: complete). The total score of COVES is 6, and poor cortical venous outflow was defined as COVES = 0 [13]. We classified the arterial collaterals on admission into two grades (good–intermediate and poor) according to the criteria of Menon et al. [28].

### 2.3. Neurological Outcomes

Following the European Cooperative Acute Stroke Study criteria, we classified HT into hemorrhagic infarction (HI) 1, HI2, parenchymal hemorrhage (PH) 1, or PH2. In the follow-up NCCT, any HT related to an increase of ≥4 points on NIHSS was considered symptomatic intracranial hemorrhage (sICH) [29]. We measured the farthest point on the septum, perpendicular to the ideal midline, which joined the most anterior and posterior visible points on the falx. According to the distance of the septum perpendicularly shifted to the healthy side, midline shift was classified into four grades: 0–2 mm, 2–5 mm, 5–10 mm, and >10 mm [30]. An mRS score of ≥3 at discharge was classified as a poor clinical outcome.

### 2.4. Statistical Analyses

We used the κ coefficient to assess interobserver agreement for TS hypoplasia, IJV stenosis, arterial collaterals, HT, and midline shift. Normally distributed continuous variables are shown as means ± standard deviations (SDs), and non-normally distributed continuous variables are presented as medians and interquartile ranges (IQRs). Frequencies (percentages) are used to describe categorical variables. According to the normality of the distribution, Student’s *t*-test or the Mann-Whitney U test was used to compare the differences between groups of continuous variables. We used the χ2 test and Fisher’s exact test to compare dichotomous variables between groups, and we used the Kruskal-Wallis H test for ordered categorical variables. Independent outcome predictors were identified by multiple binary logistic regression analyses. In the univariate analysis, any covariates with a *p*-value of ≤0.1 were entered into the logistic regression model. The favorable outflow of the affected IJV and bilateral IJVs were entered into the model, respectively. Results are presented as odds ratios (ORs) and 95% confidence intervals (CIs). Results were considered statistically significant when the two-tailed *p*-value was <0.05 (SPSS for Windows, Version 22.0; IBM, Armonk, NY, USA).

## 3. Results

### 3.1. Patient Characteristics

**Figure 1:**
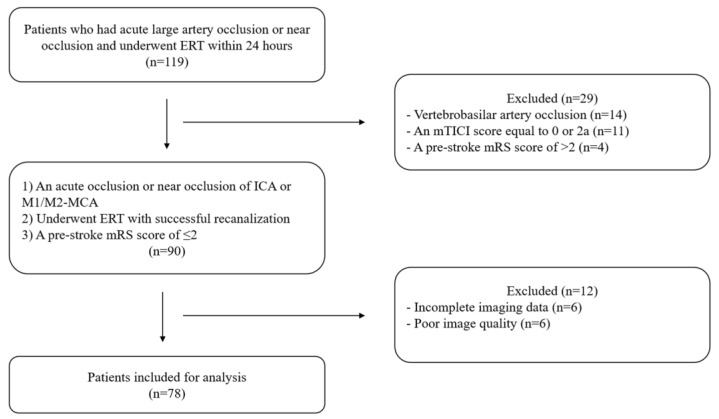
Flow chart. ERT: endovascular reperfusion therapy, mTICI: modified thrombolysis in cerebral ischemia, mRS: modified Rankin Scale, ICA: internal carotid artery, MCA: middle cerebral artery.

We identified 119 patients with acute large artery occlusion or near occlusion who underwent ERT within 24 h and excluded 41 patients (Figure 1). Finally, we examined 78 patients (near occlusion: n = 5). 

The interobserver agreements (κ) for TS hypoplasia, IJV stenosis, COVES, arterial collaterals, HT, and midline shift were 0.91, 0.90, 0.71, 0.87, 0.92, and 0.83, respectively. Table 1 presents the clinical and radiological characteristics of all patients at baseline. Among these patients, the mean age was 68 years, and twenty-seven patients (34.6%) were female. Twenty-eight patients (35.9%) had a favorable outflow of IJV on the affected side. Women were more likely to have a favorable outflow of IJV, and there was no significant difference in other characteristics among groups.

### 3.2. Association between the Outflow of TS and IJV on the Affected Side and Outcomes

There was no significant difference between ipsilateral TS outflow and imaging and clinical outcomes (Table S1). Patients with a poor outflow of IJV on the affected side had a higher incidence of HT (32.1% vs. 52.0%, *p* = 0.091). After adjusting to the admission NIHSS and arterial collaterals, the poor outflow of IJV on the affected side was an independent predictor of HT (odds ratio [OR], 3.708; *p* = 0.024). Moreover, patients with a poor outflow of IJV on the affected side had numerical trends of higher sICH rates (3.6% vs. 18.0%, *p* = 0.140), poor clinical outcomes (51.7% vs. 72.0%, *p* = 0.182), and in-hospital mortality (0.0% vs. 10.0%, *p* = 0.154); however, statistical differences were not observed. Table 2 and Table 3 present detailed information about these associations.

### 3.3. Association between the Outflow of Bilateral IJVs and Outcomes

**Figure 2:**
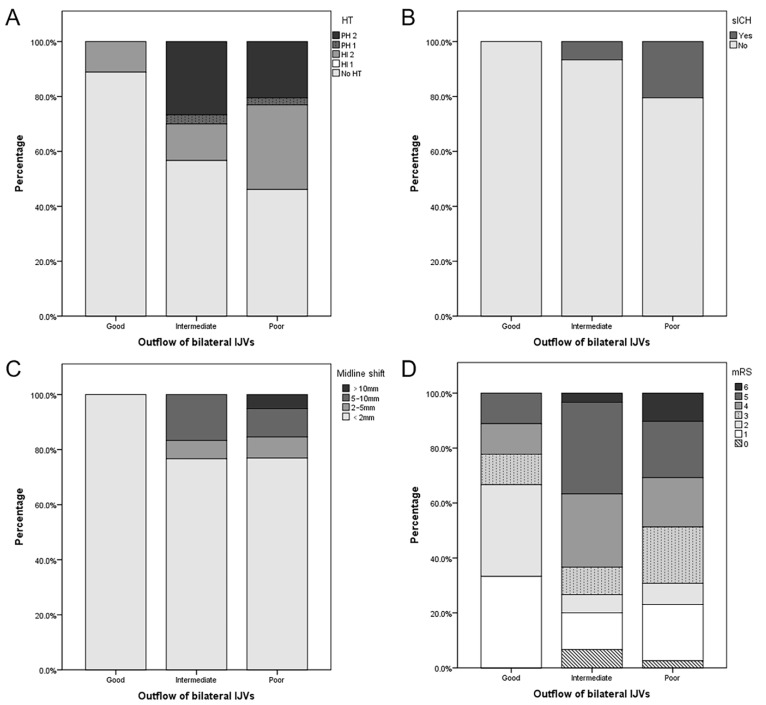
Association between imaging and clinical outcomes and the outflow of bilateral internal jugular veins (IJVs). Patients with a favorable outflow of bilateral IJVs were less likely to undergo hemorrhagic transformation (HT) (**A**), symptomatic intracranial hemorrhage (sICH) (**B**), severe midline shift (**C**), and poor functional outcome (**D**).

**Figure 3:**
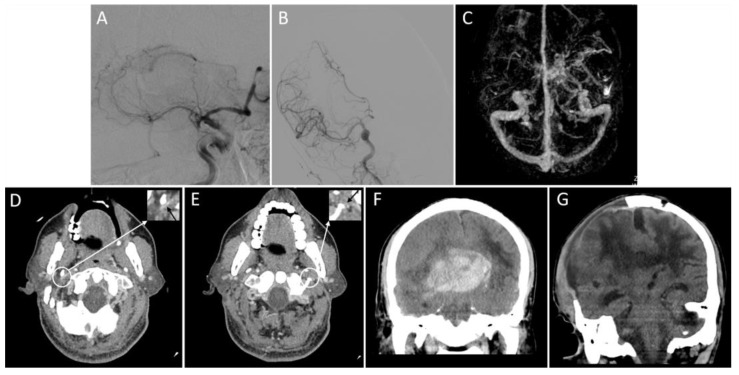
Representative images of the outflow profile of internal jugular veins and outcomes. An adult patient with an acute right middle cerebral artery occlusion (**A**) was admitted with a National Institutes of Health Stroke Scale score of 19. The patient received a thrombectomy for 6 h following stroke onset and achieved successful reperfusion (modified thrombolysis in cerebral infarction score = 3) (**B**). Three-dimensional reconstructed computed tomography (CT) venography shows that the right transverse sinus is larger than the left (**C**), and bilateral internal jugular veins are severely narrowed on the axial CT angiography (**D**,**E**, black arrows). On the non-contrast CT scan within 24 h after thrombectomy, a parenchymal hematoma, midline shift, and subfalcine hernia are present (**F**). Sixteen days after thrombectomy (15 days after decompressive craniectomy), there is an apparent cerebral edema (**G**). The patient’s modified Rankin Scale score at discharge is 5.

As shown in Table S2 and Figure 2, patients with a poor outflow of bilateral IJVs had a lower baseline NIHSS (17 vs. 12 vs. 11, *p* = 0.010), a longer onset-to-puncture time (4.0 vs. 6.2 vs. 8.0, *p* = 0.013), and a lower proportion were females (66.7 vs. 50.0 vs. 15.4%, *p* = 0.001). They also had the numerical trends of a higher incidence of HT (11.1 vs. 44.3 vs. 51.3%, *p* = 0.094), PH (0.0 vs. 30.0 vs. 23.1%, *p* = 0.080), sICH (0.0 vs. 6.7 vs. 20.5%, *p* = 0.114), midline shift >10 mm (0.0 vs. 0.0 vs. 5.1%, *p* = 0.278), poor clinical outcomes (23.3 vs. 73.3 vs. 69.2%, *p* = 0.076), and in-hospital mortality (0.0 vs. 3.3 vs. 10.3%, *p* = 0.363), but no statistical significance was evident. Multivariate analysis of HT was not performed because of the small number of positive patients in each group. Multivariate analysis showed that poor outflow of bilateral IJVs was an independent risk factor for the poor clinical outcome (OR, 17.843; *p* = 0.006) (Table 4). A representative case is shown in Figure 3.

### 3.4. Association between the Outflow of IJV and Cortical Venous Outflow

Three patients with unclear original images of head CTA were deleted. Patients with poor IJV outflow had a higher incidence of COVES = 0 (IJV on the affected side: 17.9 vs. 53.2%, *p* = 0.003; bilateral IJVs: 11.1 vs. 21.4 vs. 60.5%, *p* = 0.001). After adjusting for sex, poor IJV outflow was still an independent risk factor for COVES = 0 (IJV on the affected side: OR, 3.721; *p* = 0.021; bilateral IJVs: OR, 5.622; *p* = 0.002) (Table 5). In this cohort, although the patients with poor cortical venous outflow had a numerical trend of a higher incidence of HT and poor clinical outcomes, there was no observed statistical difference (HT, 53.3 vs. 40%, *p* = 0.256; mRS score ≥ 3, 73.3 vs. 60%, *p* = 0.235).

## 4. Discussion

This study found that poor outflow of IJV on the affected side is an independent predictor for HT, and poor outflow of bilateral IJVs is an independent risk factor for poor clinical outcomes. Patients with a poor outflow of bilateral IJVs also had the numerical trends of a higher incidence of symptomatic intracranial hemorrhage, midline shift > 10 mm, and in-hospital mortality, but statistical significance was not observed. We also found that poor IJV outflow was independently associated with poor cortical venous outflow, which has proved to be an independent risk factor for poor prognosis after ERT [13,14,15]. These results are independent of the arterial collaterals, indicating that the increased cerebral blood flow that enters the brain tissue from the successfully reopened artery and passes smoothly through the venous end to ensure the balance between arterial and venous systems may be very important for a good prognosis in patients who underwent ERT. 

Our study shows that patients with poor IJV outflow have a higher incidence of HT. In patients with a favorable outflow of bilateral IJVs, not only the incidence of HI was lower, but also no one developed PH. These are consistent with the results of the study by Winkelmeier et al. [14] in which they reported that unfavorable cortical venous outflow increased the risk of HT. The pathology of HT is likely multifactorial and thus undefined [31]. Injured cerebral autoregulation due to large artery occlusion would cause more blood than usual to enter the brain after successful recanalization and increased blood–brain barrier permeability [32,33]. On the one hand, poor venous outflow might aggravate the cerebral autoregulation impairment, and on the other hand, it might limit the drainage of increased arterial blood and raise venous pressure, which would all further aggravate blood–brain barrier injury and cause HT [7,8,9,20,34]. 

Our study’s results are not statistically significant despite a numerical trend of the proportion of higher sICH in patients with poor IJV outflow. Hence, this could be attributed to the small number of patients recruited in this study. This is also similar to the results of the study by Winkelmeier et al. [14] in which multivariate analysis of sICH was not performed because of the small number of sICH patients. However, their study found that even non-sICH HT can have a negative effect on long-term prognosis. This is consistent with the results of some other studies that found that the occurrence of HI after ERT was also related to poor clinical outcomes [35,36,37]. These findings suggest that although some previous studies [38] have found that mild reperfusion bleeding indicates successful reperfusion and favorable functional outcomes, the potential effects of mild reperfusion bleeding after ERT on functional outcomes still need to be further studied. Additionally, in clinical practice, mild reperfusion bleeding would also affect clinicians’ judgment on the use of antithrombotic drugs.

Our study also shows that patients with a poor outflow of bilateral IJVs had a significantly worse functional prognosis than those with a favorable outflow of bilateral IJVs. This is similar to the results of Jansen et al. [13] and Faizy et al. [15] whom both reported the association between absent opacification of superficial cerebral veins and no benefit from intra-arterial therapy for patients receiving ERT. However, in the studies of stroke patients without ERT, the relationship between TS–IJV and stroke outcomes is contradictory. Yu et al. [19] and Volny et al. [24] showed that dysplasia or occlusion of the ipsilateral TS–IJV was associated with severe cerebral edema. Conversely, Puetz et al. [39] reported that abnormal TS–IJV outflow was not associated with poor functional outcomes. In addition to the differences in the study population, the lack of bilateral IJV analysis may be one of the reasons. When one side of the TS–IJV is narrow, the contralateral side can be partially compensated [40]. Therefore, bilateral IJV can reflect the overall venous outflow more sensitively than unilateral IJV. In our study, both outflows of ipsilateral IJV and bilateral IJVs were associated with HT, but only bilateral IJV outflow was related to functional prognosis.

Another important finding of our study was that poor outflow of IJV is significantly associated with the poor cortical venous outflow. COVES is the main imaging scoring method for evaluating cortical venous outflow. A number of recent large clinical studies using COVES have shown that poor cortical venous outflow was associated with poor baseline arterial collaterals, complications, and poor functional prognosis after ERT [13,14,15]. However, the mechanism of poor cortical venous outflow is not clear. One possible cause is thrombosis in arterioles and venules after arterial occlusion because patients who received intravenous thrombolysis before examination have a lower proportion of poor cortical venous outflow [15]. As mentioned above, the vast majority of cortical veins are drained to IJV through the venous sinus. Poor IJV outflow can lead to increased venous pressure downstream of cortical veins, which hinders the clearance of emboli in arterioles and venules [5]. Our results supported the possibility that poor outflow of IJV could be a promising therapeutic target after ERT, as clinical practices have shown that relieving large venous outflow tract obstruction can significantly reduce cerebral venous pressure and intracranial pressure and relieve severe brain edema [20,22,23].

Additionally, there was an interesting difference between puncture onset and poor IJV outflow. However, as we mentioned above, most of the causes of IJV poor outflow are because of the muscles and bones whose anatomical positions appear difficult to change in a short time [9,19,20,21]. In contrast, the outflow of cortical veins is more likely to change over time. Therefore, a more extensive study with a broader time from onset to CTA is needed to assess whether the outflow profiles of cortical veins and IJV, their effects on prognosis change over time, and the factors that affect the outflow changes.

There are several limitations to our study. First, this was a retrospective single-center study with a relatively small sample size, causing inevitable selection bias and a relatively low lower confidence limit for bilateral IJVs in multivariate analysis. Nonetheless, the strict inclusion and exclusion criteria make the interpretation of the results more targeted. Second, we included five cases of ICA or MCA near occlusion because the hemodynamic changes before and after the lesions detected by ultrasound were almost the same in patients with ICA occlusion and near occlusion [41]. However, if the sample size is large enough, they should be analyzed separately to generate a more targeted interpretation of the results. Third, some patients may have been identified as HT from contrast staining rather than an actual hemorrhage, explaining the higher proportion of patients with HT in this cohort. However, Renu et al. [42] found that both contrast staining and hemorrhage (both blood–brain barrier disruptions) were associated with poor outcomes for stroke patients who received endovascular treatment. In addition, isolated contrast staining was related to delayed HT. Fourth, the clinical outcome was observed over a short period of time. However, given that the neurological function of patients with acute large artery occlusion may rapidly aggravate after ERT, hospitalization is a key period that requires close attention [12]. In the future, prospective studies with larger sample sizes and longer follow-up times are needed to confirm our results.

## 5. Conclusions

Patients with acute ICA or MCA stroke who experience successful recanalization following endovascular treatment have a higher risk of poor outcomes if the outflow of IJV is poor, especially on both sides. What’s more, a poor outflow of IJV is an independent determinant of poor cortical venous outflow. A larger prospective study with an extended observation period is necessary to confirm these results.

## Figures and Tables

**Table 1:** Clinical and imaging characteristics of the study cohort classified by the outflow of transverse sinus and internal jugular vein on the affected side.

Variable	Total (n = 78)	IJV Outflow		
		Favorable (n = 28)	Poor (n = 50)	*p* Value
Age (year) (IQR)	68 (60–76)	67 (59–76)	70 (61–77)	0.501
Female sex (%)	27 (34.6)	14 (50.0)	13 (26.0)	0.033
Hypertension (%)	46 (59.0)	13 (46.4)	33 (66.0)	0.092
Diabetes mellitus (%)	19 (24.4)	7 (25.0)	12 (24.0)	0.921
Hyperlipidemia (%)	12 (15.4)	2 (7.1)	10 (20.0)	0.237
Atrial fibrillation (%)	32 (41.0)	13 (46.4)	19 (38.0)	0.468
Previous stroke (%)	15 (19.2)	5 (17.9)	10 (20.0)	0.818
Smoking (%)	28 (35.9)	9 (32.1)	19 (38.0)	0.605
NIHSS admission (SD)	12 (5.0)	14 (5.8)	11 (4.6)	0.061
Location of occlusion				
ICA (%)	19 (24.4)	8 (28.6)	11 (22.0)	0.153
M1-MCA (%)	53 (67.9)	20 (71.4)	33 (66.0)	
M2-MCA (%)	6 (7.7)	0 (0.0)	6 (12.0)	
Poor arterial collaterals	14 (17.9)	4 (14.3)	10 (20.0)	0.528

Variables are presented as means ± standard deviations (SD), medians, and interquartile ranges (IQRs), or N (%). IJV, internal jugular vein; NIHSS, National Institutes of Health Stroke Scale; ICA, internal carotid artery; MCA, middle cerebral artery.

**Table 2:** Procedural characteristics, follow-up imaging, and clinical outcomes of the study cohort classified by the outflow of transverse sinus and internal jugular vein on the affected side.

Variable	Total (n = 78)	IJV Outflow		
		Favorable (n = 28)	Poor (n = 50)	*p* Value
**Procedural characteristics**				
Intravenous thrombolysis (%)	17 (21.8)	8 (28.6)	9 (18.0)	0.278
Onset-to-puncture (hour) (IQR)	6.5 (4.0–10.5)	4.7 (3.1–8.0)	8.2 (4.9–11.9)	0.005
Procedure time (min) (IQR)	90 (70–120)	90 (60–108)	90 (73–122)	0.401
mTICI score				
2b (%)	17 (21.8)	5 (17.9)	12 (24.0)	0.528
3 (%)	61 (78.2)	23 (82.1)	38 (76.0)	
**Follow-up imaging**				
HT (%)	35 (44.9)	9 (32.1)	26 (52.0)	0.091
HI1 (%)	0 (0.0)	0 (0.0)	0 (0.0)	0.208
HI2 (%)	17 (21.8)	3 (10.7)	14 (28.0)	
PH1 (%)	2 (2.6)	0 (0.0)	2 (4.0)	
PH2 (%)	16 (20.5)	6 (21.4)	10 (20.0)	
sICH (%)	10 (12.8)	1 (3.6)	9 (18.0)	0.140
Midline shift				
0–2 mm (%)	62 (79.5)	22 (78.6)	40 (80.0)	0.941
2–5 mm (%)	5 (6.4)	2 (7.1)	3 (6.0)	
5–10 (%)	9 (11.5)	4 (14.3)	5 (10.0)	
>10 mm (%)	2 (2.6)	0 (0.0)	2 (4.0)	
**Clinical outcomes**				
mRS score (IQR)	4 (2–5)	3 (1–5)	4 (2–5)	0.461
mRS score ≥ 3 (%)	52 (66.7)	16 (57.1)	36 (72.0)	0.182
In-hospital mortality (%)	5 (6.4)	0 (0.0)	5 (10.0)	0.154

Variables are presented as means ± standard deviations (SD), medians, and interquartile ranges (IQRs), or n (%). IJV, internal jugular vein; mTICI, modified thrombolysis in cerebral ischemia; HT, hemorrhagic transformation; HI, hemorrhagic infarction; PH, parenchymal hemorrhage; sICH, symptomatic intracranial hemorrhage; mRS, modified Rankin Scale.

**Table 3:** Multivariable logistic regression to predict hemorrhagic transformation on follow-up imaging in the study cohort.

Independent Variables	Univariate Analysis	Multivariate Analysis		
	*p* Value	OR	95% CI	*p* Value
NIHSS admission	0.003	1.197	1.071–1.338	0.002
Poor arterial collaterals	0.027	-	-	0.123
Poor outflow of IJV on the affected side	0.091	3.708	1.187–11.683	0.024

IJV, internal jugular vein; NIHSS, National Institutes of Health Stroke Scale; OR, odds ratio; CI, confidence interval.

**Table 4:** Multivariable logistic regression analysis to predict poor clinical outcome (mRS score of ≥3) in the study cohort.

Independent Variables	Univariate Analysis	Multivariate Analysis		
	*p* Value	OR	95% CI	*p* Value
NIHSS admission	0.021	1.220	1.073–1.386	0.002
Onset-to-puncture	0.033	-	-	0.077
Diabetes mellitus	0.062	-	-	0.145
Outflow of bilateral IJVs	0.076	-	-	-
Favorable	-	Reference	-	-
Intermediate	-	19.846	2.531–155.618	0.004
Poor	-	17.843	2.330–136.666	0.006

mRS, modified Rankin Scale; IJV, internal jugular vein; NIHSS, National Institutes of Health Stroke Scale; OR, odds ratio; CI, confidence interval.

**Table 5:** Univariate and multivariate analysis to predict poor cortical venous outflow (COVES = 0) in the study cohort ^§^.

Outflow of IJV	Univariate Analysis			Multivariate Analysis *		
	COVES = 0	COVES > 0	*p* Value	OR	95% CI	*p* Value
IJV on the affected side						
Favorable	5 (17.9)	23 (82.1)	0.003	Reference		
Poor	25 (53.2)	22 (46.8)		3.721	1.160–11.928	0.027
Bilateral IJVs						
Favorable	1 (11.1)	8 (88.9)	0.001	Reference		
Intermediate	6 (21.4)	22 (78.6)		12.267	1.389–108.325	0.024
Poor	23 (60.5)	15 (39.5)		5.622	1.848–17.105	0.002

Variables are presented as N (%). ^§^ Three cases with unclear original images of head CTA were deleted. * Adjusted for sex. IJV, internal jugular vein; COVES, cortical vein opacification score; OR, odds ratio; CI, confidence interval.

## Data Availability

The data presented in this study are available on request from the corresponding author. The data are not publicly available because it contains information that could identify the patients.

## Supplementary Materials

The following supporting information can be downloaded at: https://www.mdpi.com/article/10.3390/brainsci13010032/s1, Table S1: Clinical and imaging characteristics at baseline and post-reperfusion of the study cohort classified by the outflow of transverse sinus. Table S2: Clinical and imaging characteristics at baseline and post-reperfusion of the study cohort classified by the outflow of bilateral internal jugular veins.
