# Supplementary material for: Poor Internal Jugular Venous Outflow Is Associated with Poor Cortical Venous Outflow and Outcomes after Successful Endovascular Reperfusion Therapy
Source: Brain Sci. 2022 Dec 23;13(1):32. doi: 10.3390/brainsci13010032 (PMC9856844; doi:10.3390/brainsci13010032)
Supplement: Supplementary file 1 [file brainsci-13-00032-s001.zip › brainsci-2073302-supplementary.pdf]

Supplemental table S1. Clinical and imaging characteristics at baseline and post-reperfusion of the study cohort classified by the outflow of transverse sinus.

Variable	TS		
	No hypoplasia (*n* = 52)	Hypoplasia (*n* = 26)	*p* value
Age (year) (IQR)	68(59–79)	70(63–73)	0.668
Female sex (%)	16(30.8)	11(42.3)	0.313
Hypertension (%)	29(55.8)	17(65.4)	0.416
Diabetes mellitus (%)	14(26.9)	5(19.2)	0.456
Hyperlipidemia (%)	9(17.3)	3(11.5)	0.739
Atrial fibrillation (%)	23(44.2)	9(34.6)	0.416
Previous stroke (%)	12(23.1)	3(11.5)	0.223
Smoking (%)	21(40.4)	7(26.9)	0.243
NIHSS admission (SD)	12(5.4)	12(4.8)	0.617
Location of occlusion			
ICA (%)	14(26.9)	5(19.2)	0.442
M1-MCA (%)	33(63.5)	20(76.9)	
M2-MCA (%)	5(9.6)	1(3.8)	
Poor arterial collaterals	10(19.2)	4(15.4)	0.917
**Procedural characteristics**			
Intravenous thrombolysis (%)	10(19.2)	7(26.9)	0.438
Onset-to-puncture (hour) (IQR)	7.0(3.8–10.5)	6.2(4.6–11.0)	0.525
Procedure time (min) (IQR)	90(70–113)	107(73–150)	0.107
mTICI score			
2b (%)	14(26.9)	3(11.5)	0.121
3 (%)	38(73.1)	23(88.5)	
**Follow-up imaging**			
HT (%)	21(40.4)	14(53.8)	0.260
HI1 (%)	0(0.0)	0(0.0)	0.061
HI2 (%)	14(26.9)	3(11.5)	
PH1 (%)	1(1.9)	1(3.8)	
PH2 (%)	6(11.5)	10(38.5)	
sICH (%)	6(11.5)	4(15.4)	0.905
Midline shift			
0–2mm (%)	41(78.8)	21(80.8)	0.916
2–5mm (%)	4(7.7)	1(3.8)	
5–10 (%)	6(11.5)	3(11.5)	
> 10mm (%)	1(1.9)	1(3.8)	
**Clinical outcomes**			
mRS score (IQR)	3(2–5)	4(1–5)	0.345
mRS score ≥3 (%)	33(63.5)	19(73.1)	0.396
In-hospital mortality (%)	2(3.8)	3(11.5)	0.326

Variables are presented as means ± standard deviations (SD), medians and interquartile ranges (IQRs), or N (%).

TS, Transverse sinus; NIHSS, National Institutes of Health Stroke Scale; ICA, Internal carotid artery; MCA, Middle cerebral artery; mTICI, Modified Thrombolysis in Cerebral Ischemia; HT, Hemorrhagic transformation; HI, Hemorrhagic infarction; PH, Parenchymal hemorrhage; sICH, Symptomatic intracranial hemorrhage; mRS, modified Rankin Scale.

Supplemental table S2. Clinical and imaging characteristics at baseline and post-reperfusion of the study cohort classified by the outflow of bilateral internal jugular veins.

Variable	Bilateral IJVs outflow			
	Favorable (*n* = 9)	Intermediate (*n* = 30)	Poor (*n* = 39)	*p* value
Age (year) (IQR)	66(61–76)	70(63–78)	68(57–76)	0.672
Female sex (%)	6(66.7)	15(50%)	6(15.4)	0.001
Hypertension (%)	3(33.3)	18(60.0)	25(64.1)	0.241
Diabetes mellitus (%)	0(0.0)	9(30.0)	10(25.6)	0.182
Hyperlipidemia (%)	1(11.1)	2(6.7)	9(23.1)	0.165
Atrial fibrillation (%)	7(77.8)	12(40.0)	13(33.3)	0.052
Previous stroke (%)	1(11.1)	5(16.7)	9(23.1)	0.648
Smoking (%)	3(33.3)	9(30.0)	16(41.0)	0.633
NIHSS admission (SD)	17(6.1)	12(4.7)	11(4.9)	0.019
Location of occlusion				
ICA (%)	2(22.2)	8(26.7)	9(23.1)	0.641
M1-MCA (%)	7(77.8)	21(70.0)	25(64.1)	
M2-MCA (%)	0(0.0)	1(3.3)	5(12.8)	
Poor arterial collaterals	1(11.1)	4(13.3)	9(23.1)	0.497
**Procedural characteristics**				
Intravenous thrombolysis (%)	3(33.3)	7(23.3)	7(17.9)	0.586
Onset-to-puncture (hour) (IQR)	4(2.7–5.5)	6.2(3.7–9.5)	8.0(4.7–12.0)	0.013
Procedure time (min) (IQR)	90(60–97)	96(76–127)	90(70–120)	0.414
mTICI score				
2b (%)	2(22.2)	6(20.0)	9(23.1)	0.945
3 (%)	7(77.8)	24(80.0)	30(76.9)	
**Follow-up imaging**				
HT (%)	1(11.1)	13(43.3)	20(51.3)	0.094
HI1 (%)	0(0.0)	0(0.0)	0(0.0)	0.080
HI2 (%)	1(11.1)	4(13.3)	12(30.8)	
PH1 (%)	0(0.0)	1(3.3)	1(2.6)	
PH2 (%)	0(0.0)	8(26.7)	8(20.5)	
sICH (%)	0(0.0)	2(6.7)	8(20.5)	0.114
Midline shift				
0–2mm (%)	9(100.0)	23(76.6)	30(76.9)	0.278
2–5mm (%)	0(0.0)	2(6.7)	3(7.7)	
5–10 (%)	0(0.0)	5(16.7)	4(10.3)	
> 10mm (%)	0(0.0)	0(0.0)	2(5.1)	

**Clinical outcomes**				
mRS score (IQR)	2(1–3.5)	4(2–5)	3(2–5)	0.181
mRS score ≥3 (%)	3(33.3)	22(73.3)	27(69.2)	0.076
In-hospital mortality (%)	0(0.0)	1(3.3)	4(10.3)	0.363

Variables are presented as means ± standard deviations (SD), medians and interquartile ranges (IQRs), or N (%).

IJV, Internal jugular vein; NIHSS, National Institutes of Health Stroke Scale; ICA, Internal carotid artery; MCA, Middle cerebral artery; mTICI, Modified Thrombolysis in Cerebral Ischemia; HT, Hemorrhagic transformation; HI, Hemorrhagic infarction; PH, Parenchymal hemorrhage; sICH, Symptomatic intracranial hemorrhage; mRS, modified Rankin Scale.
